# Developing pH-Modulated Spray Dried Amorphous Solid Dispersion of Candesartan Cilexetil with Enhanced In Vitro and In Vivo Performance

**DOI:** 10.3390/pharmaceutics13040497

**Published:** 2021-04-06

**Authors:** Surendra Poudel, Dong Wuk Kim

**Affiliations:** Vessel-Organ Interaction Research Center (VOICE, MRC), BK21 FOUR Community-Based Intelligent Novel Drug Discovery Education Unit, Research Institute of Pharmaceutical Sciences, College of Pharmacy, Kyungpook National University, Daegu 41566, Korea; surendrapoudel1016@gmail.com

**Keywords:** amorphous solid dispersion, candesartan Cilexetil, PVPK30, pH-modulation, spray drying, bioavailability

## Abstract

Candesartan cilexetil (CC), a prodrug and highly effective antihypertensive agent, is a poorly soluble (BCS Class II) drug with limited bioavailability. Here, we attempted to improve CC’s bioavailability by formulating several CC-loaded amorphous solid dispersions with a hydrophilic carrier (PVPK30) and pH modifier (sodium carbonate) using the spray drying technique. Solubility, in vitro dissolution, and moisture content tests were used for screening the optimized formulation. We identified an optimized formulation of CC/PVPK30/SC, which at the ratio of 1:0.5:1 (*w*/*w*/*w*) exhibited a 30,000-fold increase in solubility and a more than 9-fold enhancement in dissolution compared to pure CC. Solid-state characterization revealed that in pH-modulated CC amorphous solid dispersion (CCSD_pM_), CC’s crystallinity was altered to an amorphous state with the absence of undesirable interactions. Stability studies also showed that the optimized formulation was stable with good drug content and drug release under accelerated conditions of up to 4 weeks and real-time stability conditions of up to 12 weeks. Furthermore, pharmacokinetic parameters, such as *AUC* and *C_max_* of candesartan, had a 4.45-fold and 7.42-fold improvement, respectively, in CCSD_pM_-treated rats compared to those in the CC-treated rats. Thus, these results suggest that CCSD_pM_ is highly effective for increasing oral absorption. The application of these techniques can be a viable strategy to improve a drug’s bioavailability.

## 1. Introduction

Gradual shifts of drug therapeutics strategy toward targeting protein, ion channels, and synthesis/or regulation pathways have led to development of many lipophilic molecules or higher molecular weights molecules, or molecules with both properties. These lipophilic entities demonstrate low aqueous solubility, leading to erratic and limited oral bioavailability and poor therapeutic inefficacy [1,2,3]. As a result, formulation scientists face the significant challenges of improving the solubility and dissolution of lipophilic drugs in the gastrointestinal (GI) fluid. A promising way to increase a drug’s dissolution rate in GI fluids is to alter the solubility or the surface area of the dissolving drugs, or both [4]. Different drug formulation strategies, such as micro or nanoparticle formation [5,6], solid dispersions (SD) [7,8,9,10], lipids-based formulation [11], inclusion complexation [12] and prodrugs [13,14] have been used to increase the rate of drug absorption in GI tract.

SD is a promising and widely accepted technique to increase the aqueous solubility of hydrophobic drugs [15,16]. Amorphous solid dispersion (ASD), a subset of SD, deals with molecular dispersion of drug molecules in an amorphous nontoxic hydrophilic carrier or matrix. The rationale behind ASD is to transform the crystalline form of drug into an amorphous form, a high-energy state, thus reducing the energy required to break crystal lattice and enhancing dissolution [17]. Technologies such as spray drying, KinetiSol^®^ dispersing, and hot-melt extrusion give polymeric stabilized amorphous formulations. Spray drying is a popular solvent evaporation technique that atomizes a suspended or dissolved drug in a polymer solution or suspension with high pressure in a chamber with hot air to produce dried particles [18]. Commonly used polymers such as polyvinylpyrrolidone (PVP), sodium carboxymethyl cellulose (Na CMC), hydroxypropyl cellulose (HPC), and hydroxypropyl methylcellulose (HPMC) are highly water-soluble and help to increase the uptake of water in ASD. Spray drying techniques reduce particle size, whereas the hydrophilic polymer matrix provides an antiplasticization effect, stabilizing the amorphous form of a drug through its viscous properties, reducing the chemical potential of the drug and maintaining the drug’s supersaturation in the GI lumen thus, improving the solubility and dissolution of drug candidate [18,19,20].

**Figure 1:**
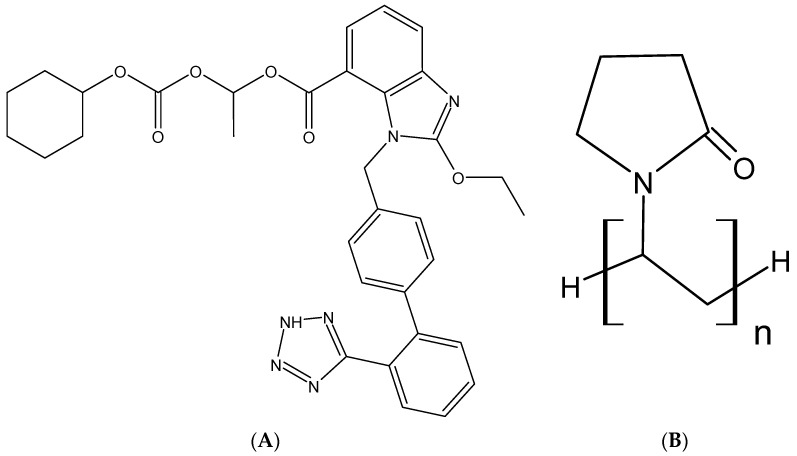

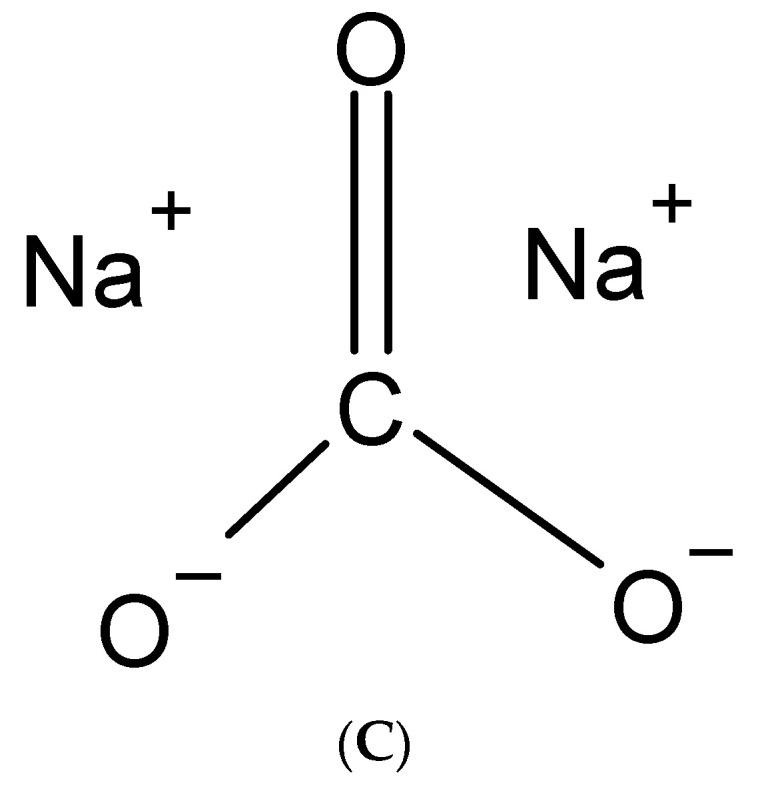
The chemical structure of candesartan cilexetil (CC) (**A**), Kollidon 30 (PVPK30) (**B**), and sodium carbonate (SC) (**C**).

Polymer’s physicochemical properties, such as melting point, glass transition temperature (T_g_), solubility, molecular weight, viscosity, and miscibility with a drug molecule play an essential role in an ASD [21]. Usually drugs with higher melting temperature (T_m_) have high lattice energy and drug with low glass transition temperature (Tg) have higher mobility. Both have high chances of crystallization and by forming ASD with polymer, the phenomena of recrystallization can be altered. The miscibility of polymer with drug decreases the T_m_ of drug while the Tg of ASD is increased [22,23]. Generally, the use of polymers with a higher glass transition temperature (Tg) in ASD increases the Tg of the dispersion system, enhancing the physiochemical stability of ASDs [24]. For example, Kollidon^®^ 30 (polyvinylpyrrolidone K30, or PVPK30; Figure 1B) is a synthetic, almost white water-soluble polymer with a T_g_ at 163 °C. It is nontoxic and used for film-forming, solubilization, stabilizing, taste masking and supersaturation maintenance/precipitation inhibitor agent [25].

Although ASD enhances the dissolution rate of hydrophobic drugs, inadequate drug solubility of polymers might result into limited enhancement. One of the commonly used strategies to maximize the aqueous solubility of weakly acidic or basic drugs is to use pH modifiers (pH_M_) with a polymer. The intraluminal pH of GI tract is diversified from highly acidic in stomach to alkaline at the intestinal regions. Such variation largely affects the weakly acidic or basic drugs as the environmental pH plays crucial role in solubility and dissolution [26]. The pH_M_ used in formulation gets dissolved in the adjacent diffusion layer and alters the microenvironment pH which induces higher saturation solubility at diffusion layer. These phenomenon leads to increased drug dissolution with supersaturation at the bulk solution. Since the supersaturation at micro-level involves the risk of rapid precipitation and/or recrystallization of drug, use of polymer helps to stabilize and maintain the supersaturation state for longer period of time which directly affects the rate of absorption [27].

Candesartan cilexetil (CC) (Figure 1A) is an ester prodrug that is generally prescribed for management of hypertension and heart failure by itself or with ACE inhibitors, beta-blockers, and diuretics. CC is biotransformed into the active metabolite, candesartan, after ester hydrolysis in the GI tract. Candesartan is a potent angiotensin II receptor blocker that restricts the activity of vasoconstrictors and produces antihypertensive effects [28]. Despite its potent therapeutic effects, CC is a BCS Class II drug with low aqueous solubility (at less than 8 × 10^−8^ M, pKa 6.0) across various physiological pH environments, contributing to its incomplete absorption in the GI tract [29,30]. CC was selected as a drug candidate because of its erratic and low bioavailability (15% to 40%) after oral administration and high first-pass metabolism. Therefore, approaches enhancing drug solubility can be handy to enhance absorption and oral bioavailability of CC. Moreover, several studies have reported enhanced candesartan bioavailability using SD [31], nano-based system [6,32,33] but increasing solubility with pH modulation in ASD is an area of interest. However, the inclusion of pH modifier comes with challenges related to production and stability due to hygroscopicity of pH modifier so, formulation with less hydroscopic pH modifier and smaller amount of pH modifier were more desired to maintain integrity of ASD.

This study aimed to design and fabricate a novel optimized CC-loaded ASD with a pH modifier to improve candesartan oral bioavailability. The CCSD_pM_ was prepared by the solvent evaporation technique with selected polymer and alkalizer in a spray dryer. The optimized formulation was characterized using solid-state characterization tools, such as scanning electron microscopy (SEM), Fourier transform infrared (FTIR) spectroscopy, differential scanning calorimetry (DSC), and X-ray diffraction (XRD). The in vitro dissolution of CCSD_pM_ was assessed in various media. Finally, the bioavailability of CCSD_pM_ was compared with pure drug CC in Sprague–Dawley rats.

## 2. Material and Methods

### 2.1. Materials

CC (Hanmi Fine Chemical Co. Ltd., Siheung, Korea), candesartan (purity > 99%; ALADDIN Biochemical Technology Co. Ltd., Shanghai, China), and Kollidon 30 (PVPK30; BASF Chemical Co., Ludwigshafen, Germany) were obtained. Sodium carbonate, sodium hydroxide, potassium hydroxide, and sodium bicarbonate were purchased from Duksan Chemicals Co. (Ansan, Korea). All other components and chemical agents were of analytical or HPLC grade; they were used without additional purification.

### 2.2. Saturation Solubility Studies of CC

The saturation solubility of candesartan cilexetil (CC) was examined in different pH (1.2, 4.0, 6.8, and 10) and deionized water. An excess amount of CC was added to an Eppendorf tube containing 1 mL of the test solution. The mixture was thoroughly vortexed and placed in an isothermal shaking water bath at 25 ± 0.5 °C at 100 rpm for five days. Then, the samples were centrifuged at 10,000× *g* for 5 min followed with filtration using a 0.45-µm membrane filter. Next, the filtrate was diluted with the deionized water to determine the drug concentration using UV spectroscopy.

### 2.3. Screening of Polymer Carrier and Alkalizers

Various polymers and alkalizers were screened with the saturation solubility method to select a suitable carrier and alkalizing agent. In short, an excess amount of CC was added to a tube containing 1% (*w*/*v*) of a polymer or an alkalizer followed by vortexing. All the samples were then incubated in a shaking water bath at 25 ± 0.5 °C and 100 rpm. After five days, the samples were centrifuged at 10,000× *g* for 5 min and filtered using a 0.45 µm membrane filter. Then, the filtrates were analyzed with a UV spectrophotometer at 254 nm.

### 2.4. Supersaturation Stabilization Assessment

Polymers are commonly known to stabilize or inhibit precipitation of supersaturated drugs [20,34,35]. A polymer’s ability to stabilize supersaturation is studied using the solvent shift method [36]. A dissolution apparatus (ERWEKA; DT 620, Heusenstamm, Germany) was the ideal instrument to maintain the temperature and rpm of the test solution. For each test solution, a selected polymer was dissolved at the concentration of 0.05% and maintained at 37 °C and 50 rpm [37]. Here,1 mL of 25 mg/mL CC in DMSO was added into a 900 mL of the test solution; 2 mL of the mixture was withdrawn with subsequent equal replacement, filtered through a 0.22 mm Millipore filter at predetermined time intervals within 240 min, and analyzed using a UV spectrophotometer (see Section 2.6).

### 2.5. Preparation of ASD

The selected polymer and alkalizing agents were used to formulate different pH-modulated ASDs to identify the optimal formulation. A lab-scale spray dryer (ADL311S; Yamato Scientific, Tokyo, Japan) was used to prepare ternary SDs of the drug, polymer, and alkalizer. At 1 g of CC in 100 mL of methanol, the methanolic solution of CC was dispersed into 200 mL of deionized water containing different amounts of carrier and alkalizer (fixed at 1 g) with stirring to produce a clear homogenous solution (Table 1). The clear solutions were then transferred at a rate of 3.5 mL/min with a peristaltic pump through a nozzle with a 0.4-mm diameter. The air was heated to 120 °C in the drying chamber and the air escaping from the spray dryer was at 65–70 °C. The atomizing was maintained at 0.15 MPa. The average flow rate of drying air was adjusted at 0.13 m^3^/min by setting the blower knob at 5.5. In total, five SDs were prepared for further evaluation to select the optimized ternary solid dispersion.

### 2.6. UV-VIS Spectroscopy

The quantitative measurement of CC was conducted with a UV-VIS spectrophotometer (UV-1800; Shimadzu, Kyoto, Japan). The standard calibration curve was drawn from known CC concentrations between 0.625 and 25 μg mL^−1^ in methanol measured at 254 nm using Beer–Lambert plots. A standard curve with linearity (*R*^2^ = 0.9991) was used for the quantification of CC.

### 2.7. Optimization of CCSD_pM_

#### 2.7.1. Aqueous Saturated Solubility and In Vitro Dissolution Study

The solubility of the five CCSD_pM_ formulations was examined to select the formulation with maximal solubility. An excess amount of an CCSD_pM_ was added to a vial containing 1 mL of deionized water. The resultant suspension was maintained at 25 ± 0.5 °C and 100 rpm in an isothermal shaking water bath for five days. Next, the CCSD_pM_ suspension was centrifuged at 10,000× *g* for 5 min and filtered to obtain a clear supernatant solution. The drug’s concentration in the supernatant was determined with a UV spectrophotometer at 254 nm.

The in vitro dissolution behavior is one of the crucial criteria in determining ASD technique viability. The dissolution test of CCSD_pM_ equivalent to 8 mg of CC and 8 mg of pure CC was performed using a USP Type II dissolution apparatus (ERWEKA; DT 620, Heusenstamm, Germany). The CCSD_pM_ or the pure CC powder was placed in a capsule (size “0”), and a sinker was used to sink the capsule to the bottom of vessels filled with 900 mL of distilled water maintained at 37 ± 0.5 °C with continuous stirring at 50 rpm. Two mL of the medium was sampled at predetermined time intervals at 5, 10, 15, 30, 45, and 60 min; after each sampling, 2 mL of fresh medium was added to replenish the volume loss. The sampled medium was filtered through 0.45 μm PTFE syringe filter, and the drug released into the medium was analyzed using a UV spectrophotometer, as mentioned above.

#### 2.7.2. Moisture Content Measurement

The moisture content of the formulations F1–F5 was analyzed by a moisture analyzer (OHAUS, MB 120). The instrument records the difference in the sample’s weight before and, after heating up to 100 °C for 10 min. Finally, the difference in weight was displayed as percentage of moisture content (MC), an indicator of water content in the sample.

### 2.8. Characterization of CCSD_pM_

#### 2.8.1. Drug Content Assay

The estimation of drug loading is crucial to exclude drug loss by the spray drying process. For drug loading assay, 100 μg/mL of CC in methanol was prepared with the equivalent amount of CCSD_pM_, filtered, and analyzed using UV spectroscopy (Section 2.6). The mathematical relation, drug content (%) = estimated drug content/theoretical drug content × 100, was used to determine drug content.

#### 2.8.2. External Morphology

The surface morphology of pure drug, polymer, alkalizer, physical mixture (PM), and optimized CCSD_pM_ was assessed using a scanning electron microscope (SEM) (SU8220; Hitachi; Tokyo, Japan). The physical mixture was composed of CC, Kollidon 30, and Na_2_CO_3_ in the weight ratio of 1:0.5:1. The samples were fixed on the brass post with two-sided adhesive tapes and sputter-coated for 4 min at 5.0 kV and 15 mA using a Sputter Coater (EMI Tech, K575 K; West Sussex, UK).

#### 2.8.3. Differential Scanning Calorimetry (DSC)

The change in the physical properties of CC, Kollidon 30, Na2CO3, PM, and CCSD_pM_, F1, along with temperature against time, was measured using a thermal analysis instrument (DSC Q20; Newcastle, DE, USA). Approximately 5 mg of a sample was placed in a non-hermetically enclosed aluminum pan and heated with dry nitrogen purge at 50 mL/min. The instrument was operated at a scan rate of 10 °C/min in a heating range of 30–220 °C.

#### 2.8.4. X-ray Diffraction (XRD)

The X-ray diffraction (XRD) analysis of the pure drug, polymer, alkalizer, PM, and CCSD_pM_ was performed using a D/MAX-2500 XR instrument (Rigaku, Japan) equipped with a Cu anode. The X-ray beam was operated at a voltage of 40 kV and a current of 40 mA. The scanning range was between 5°–35° (2θ) with a scanning speed of 0.05°/s. The diffractograms was plotted using the SIGMA PLOT 12.0 software.

#### 2.8.5. Fourier Transform Infrared Spectroscopy (FTIR)

The percentage transmittance of the pure drug, polymer, alkalizer, PM, and F1 was analyzed with an FTIR spectrophotometer (Frontier; PerkinElmer, Norwalk, CT, USA). The samples were placed with a KBr disk and scanned from 4000 to 400 cm^−1^ at a 2 cm^−1^ resolution. The KBr pellets were a mixture of 1 g of sample and 200 mg of KBr.

### 2.9. Stability Assessment

The optimized pH-modulated ASD (F1) was investigated for the changes in its attributes over time under environmental conditions such as temperature and humidity. As per ICH guidelines, the samples were introduced into a stability chamber maintained at real-time (RT) at 25 ± 2°C, 60 ± 5% relative humidity (RH) and the accelerated stability conditions (ACC) of 40 ± 2 °C and 75 ± 5% RH [38]. At predetermined intervals, 1, 4, 8, and 12 weeks for RT and 1 and 4 weeks for ACC, the samples were analyzed for drug content and in vitro dissolution by UV spectroscopy.

### 2.10. Pharmacokinetic Study

#### 2.10.1. Animal Handling and Blood Sampling

Sprague Dawley rats aged 7–9 weeks and weighing 280–343 g, were used for the in vivo experiments. The rats were adapted to the controlled conditions of 25 ± 2 °C, 55 ± 5% RH, and 12-h light/dark cycles for at least 3 days. The rats were abstained from food and had free access to water for at least 12 h ahead of the oral administration of the drugs. All the procedures were as per established codes at Kyungpook National University (Institutional Animal Care and Use Committee, License Number: 2019-0054, Authorization date: 1 March 2019).

Twelve rats are divided into two groups, with 6 per group (*n* = 6). Each rat was given 10 mg/kg CC aqueous suspension or F1 (the optimized CCSD_pM_) at a single dose with oral gavage. The CC aqueous suspension was developed by dispersing the pure CC powder in 0.5% *w*/*v* in Na CMC. Subsequently, approximately 0.3 mL of the blood sample was collected in a heparinized tube at predetermined intervals of 0.083, 0.25, 0.5, 1, 2, 4, 8, 12, and 24 hr. Juglar vein was selected for blood sampling after the rats were anesthetized with diethyl ether. The collected blood samples were immediately centrifuged at 13,000× *g* for 10 min at 4 °C and stored at −20 °C.

#### 2.10.2. Plasma Sample Analysis

A 90 µL plasma sample was vortexed with 40 µL of irbesartan (IS), internal standard, and 270 µL of ACN, a precipitating agent, for a few minutes, and centrifuged at 1000× *g* for 20 min. The final concentration of IS was maintained at 1 µg/mL. The clear supernatant liquid was transferred into an HPLC vial, and 20 µL of it was injected into a column (Capcell Pak C18, 4.6 × 250 mm) equilibrated at 25 °C with a mobile phase of ACN: MeOH: Potassium dihydrogen phosphate at pH 3.0 at a ratio of 30:30:40 (*v*:*v*:*v*). The samples were injected at a flow rate of 1.0 mL/min, and the quantification was done at ʎ_max_ 254 nm. A standard plasma curve was projected in the range of 25–5000 ng/mL, showing good linearity (*R*^2^ = 0.9998).

#### 2.10.3. Statistical Evaluation

The pharmacokinetics parameters of candesartan, such as the area under the concentration-time curve (*AUC*), half-life (T_1/2_), peak plasma concentration (*C_max_*), time for *C_max_* (T_max_), and elimination-rate constant (K_el_) of each rat, were determined by fitting to a noncompartmental analysis using WinNonlin^TM^ (Pharsight Corp.; Mountain View, CA, USA). The *AUC*_0–24h_ was estimated using the trapezoidal rule. Unpaired Student’s *t*-test was used to analyze the level of statistical significance (*p* < 0.05).

## 3. Results and Discussion

### 3.1. Saturation Solubility and Screening Studies

Understanding the solubility profile of a drug in various test solutions would help select the carriers and solubilizers that are optimal for the preparation of ASDs. Polymers and other excipients must have good compatibility and affinity with the drug to achieve the desired in vitro dissolution rate, which is correlated to a favorable in vivo drug profile [9]. CC behaves like a weakly acidic drug and gets deprotonated in solutions with a high pH (pH > pKa), resulting increased solubility profile [39].

**Figure 2:**
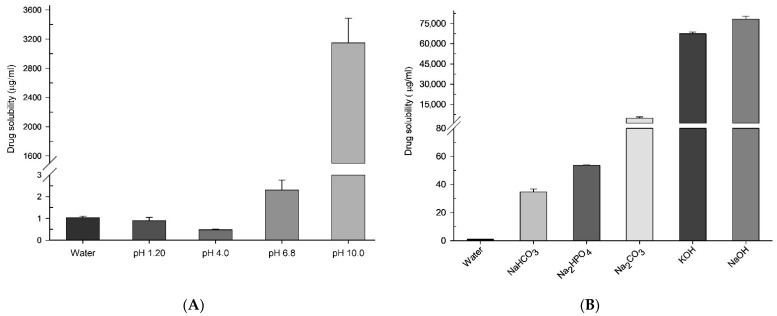
Aqueous solubility of CC in (**A**) different pH conditions and (**B**) different alkalizers (1% *w*/*v*). Each value represents the mean of three experiments (*n* = 3), and the error bar represents the standard deviation.

Among the test solutions with different pH values, CC had remarkably high solubility, at 3148.29 ± 338.32 µg/mL, at pH 10.0, and very low solubility at pH 1.2, 4.0, 6.8, and water (Figure 2A). These data demonstrate that ASD with alkaline pH modifiers will enhance CC’s solubility. In the solubility screening of the alkalizers as pH modifiers, the CC in 1% *w*/*v* sodium hydroxide solution had the highest solubility at 78,254.85 ± 2239.49 µg/mL; in contrast, the CC in sodium bicarbonate had the lowest solubility at 34.67 ± 2.03 µg/mL (Figure 2B). Though the highest solubility was demonstrated by hydroxides of sodium and potassium, sodium carbonate was chosen because the hydroxides were strong alkalizing agents with more hygroscopicity properties that would impact the stability of formulations.

**Figure 3:**
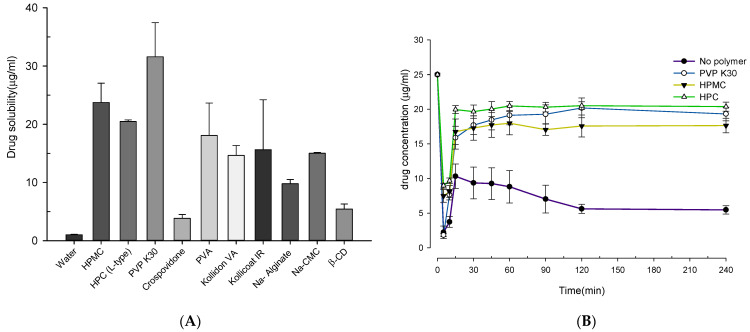
(**A**) The solubility of CC in different polymer solutions (1% *w*/*v*). (**B**) The concentration-time profile of CC in various test solutions. Each value represents the mean of three experiments (*n* = 3), and the error bar represents the standard deviation.

Similarly, the solubility study (Figure 3A) of the polymeric carriers showed that Kollidon 30 (PVPK30) had the highest drug solubility at 31.60 ± 5.84 µg/mL whereas copovidone had the lowest drug solubility at 3.83 ± 0.67 µg/mL among the hydrophilic carriers. Kollidon 30 is a hydrophilic polymer with successful pharmaceutical applications. Its amorphous nature, higher T_g_, and hydrophilicity indicate its suitability for the spray drying technique.

In addition to solubility screening, the supersaturation maintenance test was also conducted to analyze ability of polymer to stabilize or inhibit precipitate from saturated state of drug in a non-sink condition. Generally, ASD techniques generate drugs in an amorphous state that tends to precipitate rapidly in GI lumen; thus, polymeric stabilizers can extend and maintain a drug in a supersaturable state in GI lumen [40]. Here, PVPK30, HPMC, HPC, and distilled water were used as test solutions to study the supersaturation maintenance behavior for 4 h, which mimics intestinal transit. The supersaturation profile (concentration-time) of the individual polymers was examined (Figure 3B). In all the test solutions, a rapid decrease in CC concentration was observed immediately after the injection of concentrated CC; subsequently, CC concentration was maintained based on the polymer’s supersaturation maintenance ability. HPC had highest ability to maintain CC concentration of 20.38 μg/mL for up to 240 min, whereas HPMC had least ability. In addition, PVPK30 could also maintain a CC concentration of approximately 19.3 μg/mL for up to 240 min. Similar behavior can be correlated with the saturation solubility study, as polymers can increase CC’s solubility.

The stabilization of the supersaturation state is a complex phenomenon which can be regulated with increasing the solubility by reducing nucleation and crystal growths, increasing the viscosity by reducing molecular mobility, thus decreasing nucleation and crystal growth and altering the solvation level at the crystal/liquid interface [41]. A slight increase in supersaturation maintenance of CC by HPC could be related to the viscosity phenomenon by cellulose-based polymers, which in turn limits molecular mobility [42]. Considering the results from the solubility enhancement and supersaturation stabilization studies, PVPK30 was chosen for developing pH-modulated ASDs using the spray drying method.

### 3.2. Optimization of CCSD_pM_ Formulations

**Figure 4:**
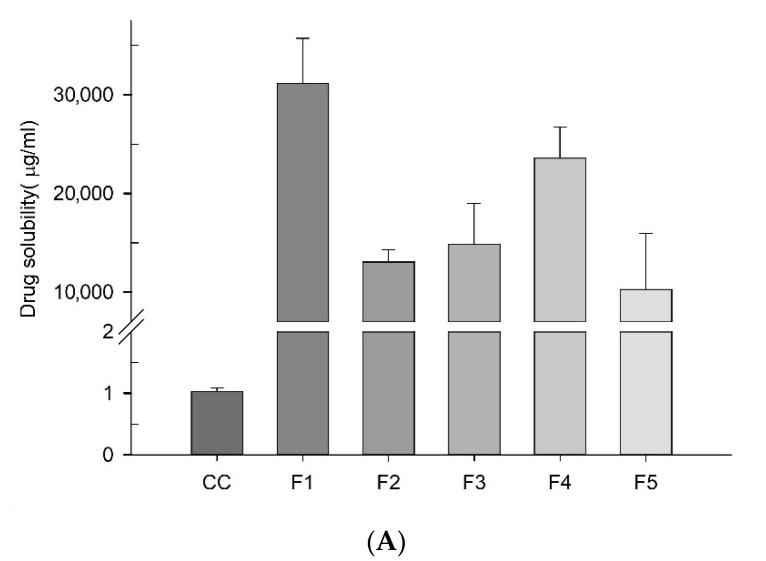

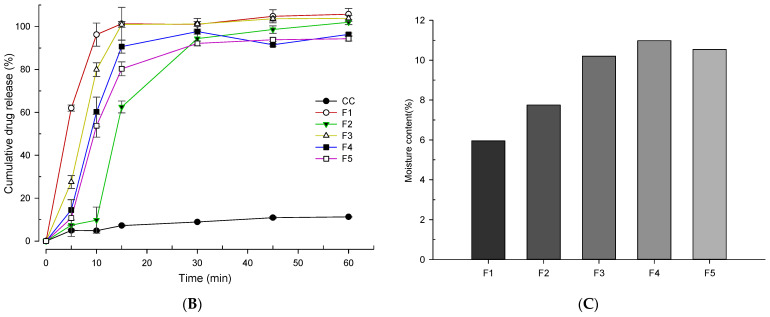
(**A**) The drug solubility of the CCSD_pM_ formulations (F1–F5). (**B**) The in vitro release profiles of the pure drug from the capsules compared to that of the different CCSD_pM_ formulations in distilled water. (**C**) The moisture content of the CCSD_pM_ formulations.

Sprayed dried CCSD_pM_ formulations (F1–F5) were prepared using a spray dryer (ADL311S; Yatamo Scientific) with PVPK30 as a hydrophilic carrier and SC as an alkalizer. The effect of varying weight ratios of PVPK30 on the solubility of a CCSD_pM_ formulation was studied by assessing the solubility profiles of all the formulations (Figure 4A). The incorporation of SC and PVPK30 demonstrated a phenomenal enhancement of CC solubility, in CCSD_pM_’s compared to the CC powder, at more than 15,000-fold, irrespective of the polymer ratios. Additionally, increasing PVPK30 did not consistently improve CC’s solubility as F1 with the lowest PVPK30 showed maximum solubility.

The degree of pH modulation with SC in varying drug: polymer ratio is an important aspect for such differences in solubility of five formulations. The maximum enhancement in F1 could be due to adequate microenvironmental pH modulation with SC in given lower amount of PVPK30. Additionally, drug particles might have extensive size reduction achieving increased surface area with molecular dispersion into given amount of PVPK30 matrix [9]. Surprisingly, F2–F4 have increasing trends in solubility and F5 shows slight reduction in solubility enhancement. The uptrend of F2–F5 suggest that, SC doesn’t have major effect as in F1 but the increasing amount of PVPK30 in presence of SC increased the solubility. However, in F5, there was not significant cumulative effect of PVPK30 and SC as amount of PVPK30 was highest. Although, there was slight reduction in solubility of F5, it still had more than 15,000-fold improvement in comparison with pure CC.

While the conventional SD system increases the solubility and dissolution profile of hydrophobic drugs, using large amount of polymer with exclusive or heavy use of organic solvent in the formulation had been a major issue. Organic solvents are not ideal from health, environmental and industrial-scale perspectives. Thus, the difference in solubility of polymers and drugs in an organic solvent and incomplete removal of organic solvents are significant limitations associate with conventional SD techniques [14,43]. We attempted to reduce the toxicity of an organic solvent by dissolving the drug in a relatively small volume of organic solvent followed with dispersion in the aqueous phase. In addition, a relatively small amount of polymer was used for solubilization in the presence of an alkalizer.

The dissolution study of the CCSD_pM_ formulations in deionized water revealed that the incorporation of SC as an alkalizer and PVPK30 as a hydrophilic carrier greatly enhances their drug release rate compared to the CC powder. The CC powder’s release rate was only about 11% at 60 min, while all the CCSD_pM_ formulations released more than 90% of their drugs at 30 min. F2 had a slower release rate than others before 30 min, while other formulations had a similar release rate (Figure 4B). The difference in the release rate of the CCSD_pM_ formulations may be due to the differences in their solubilities, water penetration time inside the capsule, and relative time for the alkalizer to maintain the alkaline microenvironment. Once CCSD_pM_ formulations were in contact with water, the alkalizer modulates the solution surrounding the CCSD_pM_ to enable CC’s transformation into an unprotonated state [44]. Besides the alkalizer, another contributing factor may be the wetting ability of the polymer along with the transformation of the crystallinity state into the amorphous state (from results of solid-state characterization).

The amount of moisture can directly affect a drug’s physical stability as well as the recrystallization phenomenon. Here, the water content of all the formulations (F1–F5) was also measured. About 10.9% moisture was observed in the F4 formulation, the highest among the ASDs. On the other hand, F1 had the lowest moisture content at about 5.96%, which was correlated to its smallest amount of hydrophilic polymer among the formulations (Figure 4C). The differences in the moisture content might be due to incomplete drying after spray drying and a varying amount of PVPK30 that attribute to different hygroscopicity in the formulations. After reviewing the results from the dissolution, solubility, and moisture content studies, F1 was selected for further characterization and assessment as it had the highest solubility, a better drug release rate, and lower moisture content.

### 3.3. Assessment of Selected CCSD_pM_ Formulations

**Figure 5:**
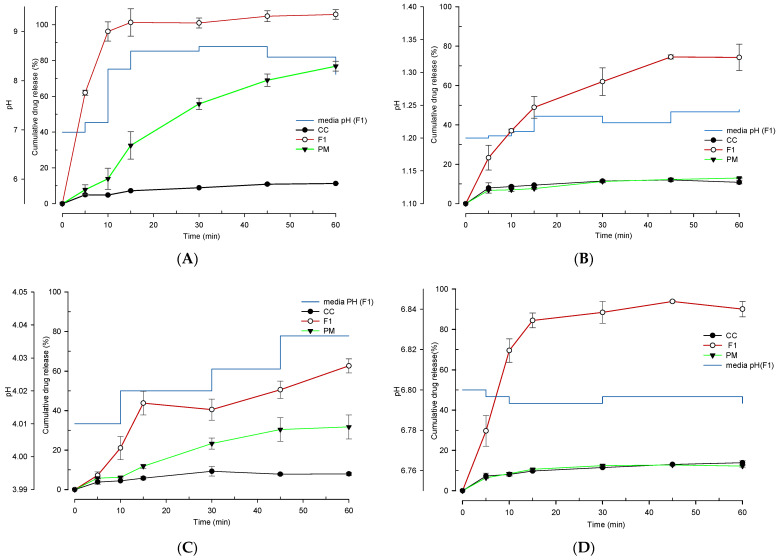
The in vitro dissolution study of CC from capsules filled with optimized formulation (F1), physical mixture (PM), and pure drug in (**A**) distilled water, (**B**) a solution at pH 1.2, (**C**) a solution at pH 4.0 and (**D**) a solution at pH 6.8. Each value represents the mean of three experiments (*n* = 3), and the error bar represents the standard deviation.

The in vitro dissolution profile of the selected CCSD_pM_, F1, was investigated at various solutions including distilled water and solutions at pH 1.2, 4.0 and 6.8, and then compared with the dissolution profile of the PM and the free CC (Figure 5). It was observed that F1 had a substantially higher drug release rate at all conditions than the PM or the free CC. At pH 1.2 and 4.0, the drug release of F1 was above 60% within 60 min; at higher pH, F1’s drug release was above 90%. On the other hand, no significant changes were observed in the release characteristics of the free CC under different conditions. However, significant variations of drug release were observed in the case of PM. There was a difference of approximately 30% in drug release at pH 4.0 solution and distilled water whereas more than a difference of 60% in drug release at pH 1.2 and 6.8 between PM and F1. The low drug release rate of PM further confirmed the superiority of CCSD_pM_ F1. The variation in drug release from F1 at different pH media solution suggest that there is an influence of pre-existing pH conditons of media (diffusion layer) in the microenvironmental pH modulation capacity of alkalizer. It was also seen that the pH of the dissolution solution was not altered drastically during the experiment, and all the dissolution profiles were correlated to the solution’s pH.

In addition, the saturated solubility of F1, PM, and free CC was studied at pH 1.2, 4.0, and 6.8 and in water (Table 2). The optimized CCSD_pM_, F1, displayed notable improvement in the saturated solubility over free CC and the PM. F1’s saturated solubility was more than 30,000-fold of that of free CC and at least 3-fold of that of PM. F1’s improvements in dissolution and solubility over the PM are due to ASDs that transform crystalline materials into amorphous forms and the solubilization, stabilization, and wetting effects of the polymers and alkalizers [44]. Further, a drug loading study showed that F1 had the highest drug content at about 99%, which could be correlated with its excellent dissolution profile.

### 3.4. Physiochemical Characterization

**Figure 6:**
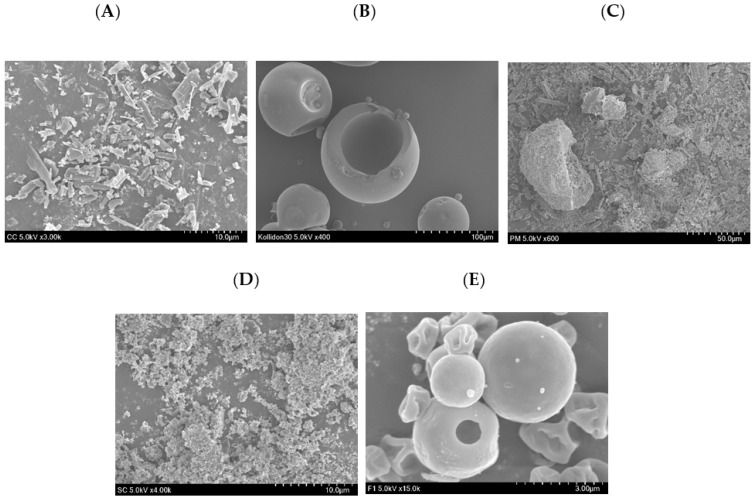
SEM image of (**A**) CC powder, (**B**) PVPK30, (**C**) SC (Na_2_CO_3_), (**D**) physical mixture (PM), and (**E**) F1.

The solid-state properties of free CC, PM, and F1 were investigated using characterization tools, such as SEM, DSC, and XRD. The external morphological view of CC, PVPK30, SC, PM, and F1 was imaged with SEM (Figure 6). The CC powder appeared as irregularly shaped crystalline structures (Figure 6A), whereas PVPK30 and SC had spherical particles with a smooth surface and fine particles with no distinctive shape, respectively (Figure 6B,C). The PM (Figure 6D) in the same ratio as F1 had the alkalizer and drug particles adhering to the carrier’s surface. Surprisingly, F1 appeared to have spherical and some dented particles without the presence of drug particles on its outer surface (Figure 6E).

**Figure 7:**
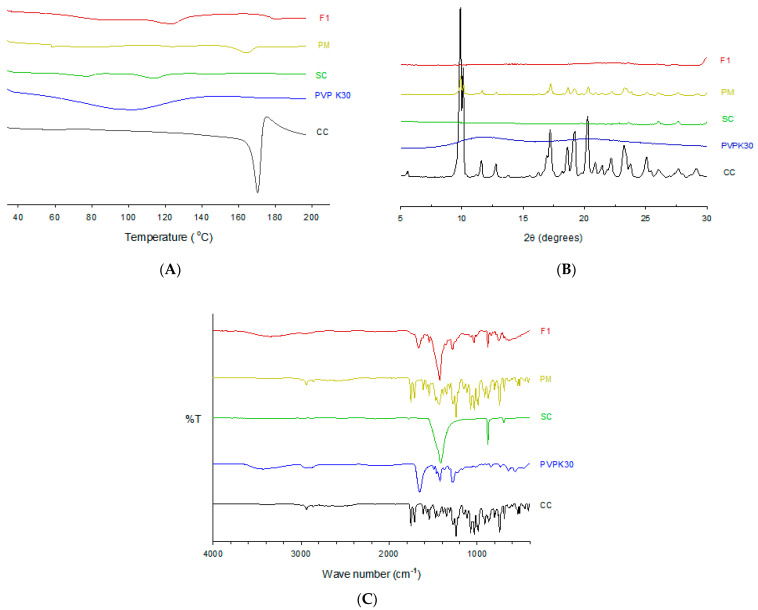
The differential scanning calorimetry (DSC) (**A**), powder X-ray diffraction (PXRD) (**B**), and FTIR (**C**) analysis of CC, PVPK30, SC, PM, and F1.

On the other hand, DSC analysis revealed the thermal characteristics of pure drug CC, PVPK30, SC, and F1 (Figure 7A). The DSC curve of CC showed an endothermic peak around 170 °C, consistent with the previous finding that CC was thermally stable at below 162 °C [45,46]. The sharp endothermic peak also confirms that CC’s structure is crystalline. Moreover, PVPK30 and SC had not shown any characteristic sharp endothermic peak except the broad endothermic peak at 50–130 °C for PVPK30 and 70–85 °C and 110–112 °C for SC. In addition, DSC of PM revealed a reduced endothermic peak of CC, suggesting weak interaction with the carriers. However, CC’s characteristic endothermic peak is absent in the F1 DSC curve, suggesting inhibited crystallinity.

The powder X-ray diffraction (PXRD) of CC, PVPK30, SC, PM and F1 were performed (Figure 7B). The diffraction pattern of CC shows numerous distinctive peaks up to 30°, corroborating with its highly crystalline nature [46]. In contrast, PVPK30 had no peaks due to its amorphous nature. Additionally, SC’s PXRD pattern showed smaller and fewer peaks, consistent with its reduced crystalline form. In addition, the PM retained intrinsic peaks of the respective ingredients with a reduced crystalline form. Lastly, the characteristic peaks of CC and SC were absent in the F1 sample, again consistent with F1’s amorphous nature.

The FTIR analysis was used to investigate the drug-polymer interaction in ASDs as it will unveil the mechanism of stabilization of ASD. The FTIR spectrum of CC, PVPK30, SC, PM, and F1 was compared (Figure 7C). The CC spectra revealed prominent peaks bands characteristic of the polymeric form I of CC [47]; the aromatic and aliphatic CH stretching in 3070–2855 cm^−1^ range with peaks at 3068, 3000, 2940, and 2860 cm^−1^; the C=O group stretching from the asymmetric organic carbonate -OC(=O)O- moiety with an intense band at 1753 cm^−1^; an ester carbonyl group at 1713 cm^−1^; the -NH bending at 1622 cm^−1^; and the aromatic C-N stretching at 1348 cm^−1^ and C-O ether stretch at 1032 cm^−1^. PVPK-30 showed distinct -C=O stretching at 1651 cm^−1^ and broad absorption bands at 3434 cm^−1^ from -OH stretching vibrations, correlating to the broad endotherm peak in the DSC study. SC’s spectrum also exhibited a broad band peaking at 1420 cm^−1^ and sharp peaks at 872 and 702 cm^−1^. Further, the PM exhibited a characteristic peak of CC and other excipients along with some superimposition. CCSD_pM_ F1’s spectra retained the characteristic peaks of CC with some enclosure suggesting weak inter-molecular H-bonding. Further examination in conjunction with other techniques, such as Raman spectroscopy and ^1^H NMR, will provide more insights into the phenomena, such as the masking and shifting in spectra.

### 3.5. Stability Studies

**Figure 8:**
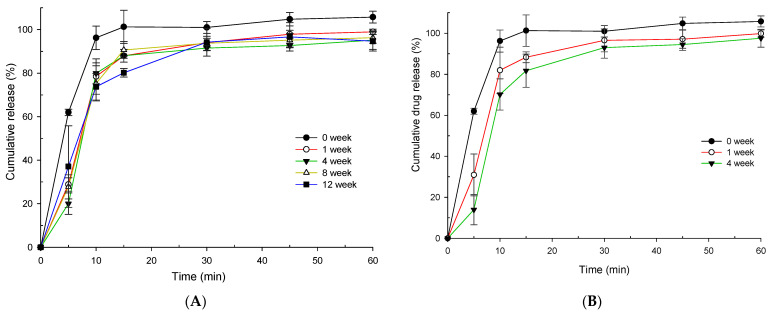
The stability study of CCSD_pM_ (F1). (**A**) The dissolution study after 1, 4, 8 or 12 weeks under real-time stability condition at 25 ± 2 °C and RH 60 ± 5%. (**B**) The dissolution study after 1 or 4 weeks under accelerated stability condition at 40 ± 2 °C and RH 75 ± 5%.

The stability study of a formulation uncovers the changes in the quality parameters under normal and stress conditions within a time frame. Different variables, such as the manufacturing process, quality of drug, excipient, and packaging material, may impact a product’s quality over time. However, the impact of heat and moisture has been identified as the principal factor determining product quality [48]. The in vitro drug release studies confirm that CCSD_pM_(F1) under real-time and accelerated stability conditions have comparable release characteristics (Figure 8). All the samples had more than 90% cumulative drug release in 30 min, indicating a consistent release profile with the 0-week sample. In addition, the drug content of F1 after 12 weeks in RTS and 4 weeks in ACC conditions was 96.38 ± 1.040 and 95.75 ± 3.53%, respectively, indicating an absence of potential degradations (Table 3).

Usually, a stability study of at least six months is required to demonstrate a product’s stability. However, in our study, the 4-week accelerated conditions and 12-real-time conditions provide a level of understanding of the effect of temperature and moisture on CCSD_pM_ formulations. Further, a formulation’s stability can be correlated with a smaller alkalizer-to-drug ratio, as high alkalizer content can give rise to instability. Though the study was of a limited time frame with only in vitro and drug content analysis, these results validate pH modulation approach to enhance absorption. Additionally, extending the study time and quantifying the impurities will further strengthen the feasibility of this approach.

### 3.6. Pharmacokinetic Studies

Candesartan is the major metabolite of CC that induces an antihypertensive effect. CC, the prodrug, is rapidly hydrolyzed to candesartan by the esterase in the gastrointestinal tract. As a result, only candesartan can be quantified in the plasma. Here the plasma concentration of candesartan at the 10 mg/kg dose for CCSD_pM_ F1 and the pure CC was successfully quantified in rats (*n* = 6) using a reverse UHPLC system.

**Figure 9:**
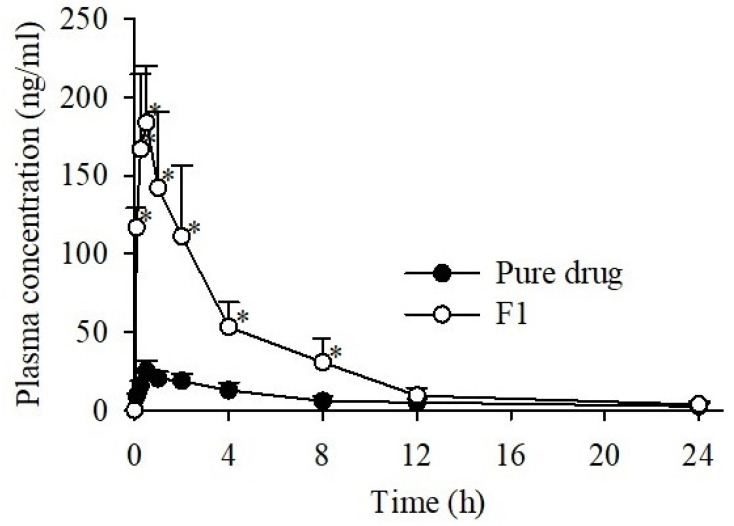
Plasma concentration-time profiles of candesartan after oral administration of pure drug (CC) or CCSD_pM_ (F1) in rats. Each value represents the mean of six experiments (*n* = 6), and the error bar represents the standard deviation. * *p* < 0.05 compared to free CC.

The mean plasma concentration-time plots and pharmacokinetic parameters of CCSD_pM_ and pure drug are summed up in Figure 9 and Table 4, respectively. As shown in Figure 9, the mean plasma concentration of candesartan from CCSD_pM_ F1 was higher than pure CC at all the time points, suggesting increased bioavailability. The maximum plasma concentration (*C_max_*) in rats administered with CCSD_pM_ was 188.75 ± 41.06 ng/mL, considerably higher, by 7.42-fold, than that of the pure CC at 25.44 ± 6.28 ng/mL. In addition, the *AUC* of candesartan from the rats administered with CCSD_pM_, at 771.87 ± 227.63 h·ng/mL, was significantly higher than that from the pure CC administration at 173.29 ± 30.27 h·ng/mL. Other pharmacokinetic parameters, such as t half (*t*_1/2_) was shorter in F1, while k elimination (K_el_) and T_max_ of CCSD_pM_ were similar in F1 and pure CC.

Altogether, these data indicate that better solubility and dissolution rate at the physiological pH range increases bioavailability, resulting in a significant enhancement in oral absorption. The rapid absorption of CCSD_pM_ can be due to its amorphous nature, increased solubility, and super-saturable state in the physiological environment. The use of polymers alone has also been accounted for increased absorption [31] but addition of pH modifiers was found to improve the overall performances significantly without any stability issues. However, the optimization of the use of alkalizers needs to be thoroughly examined with a better correlation between the in vitro and in vivo parameters. This study has taken a step to address the low bioavailability issues of weakly acidic drugs, such as CC, using polymers, alkalizers, and a spray drying technique to achieve notable improvement in the in vitro-in vivo, stability study. Furthermore, this strategy can help reduce the therapeutic dose due to the increase in bioavailability, excipients amount due to the lower carrier-to-drug ratio, and cost-effectiveness compared to the conventional methods.

## 4. Conclusions

A novel CC-loaded spray-dried ASD with pH modifiers was successfully designed and formulated to enhance candesartan’s bioavailability. The optimized formulation exhibited increased solubility, enhanced dissolution with good stability. The enhanced properties could be due to the solubilization of CC by the added polymer matrix as well as the modulation of the microenvironment pH with alkalizers. In addition, polymer PVPK30 and pH modulator sodium carbonate contributed to the maintenance of a supersaturable state, increasing the absorption potential at GI lumen. The physicochemical characterization also confirmed that CC was amorphous in the dispersed state with the polymer matrix. The pharmacokinetics of optimized CCSD_pM_ were remarkably increased compared to the pure CC, indicating better absorption and bioavailability. Although CCSD_pM_ had good stability, long-term studies are needed to reinforce and support the pragmatic shift of formulation technology. Overall, a spray-dried ASD system based on the use of a pH modifier could be a potential approach to enhance the limited bioavailability of poorly soluble drugs.

## Figures and Tables

**Table 1:** Compositions of the pH-modulated amorphous solid dispersion (ASDs) with different polymer ratios.

Formulation	CC (g)	PVPK30 (g)	Na_2_CO_3_ (g)
F1	1	0.5	1
F2	1	1	1
F3	1	2	1
F4	1	4	1
F5	1	8	1

**Table 2:** The solubility of the free CC, physical mixture (PM), and F1 in solutions with different pH.

Medium	CC (μg/mL)	PM (μg/mL)	F1 (μg/mL)
Water	1.03 ± 0.06	7613.11 ± 6480.31	31,156.05 ± 4552.69
pH 1.2	0.89 ± 0.13	10,462.60 ± 6492.75	32,212.37± 4785.75
pH 4.0	0.48 ± 0.03	103.78 ± 76.71	31,095.11 ± 5395.98
pH 6.8	2.30 ± 0.37	822.71 ± 171.25	38,424.75 ± 7539.25

Each value represents the mean of three experiments (*n* = 3) ± standard deviation.

**Table 3:** Drug content study.

Weeks	Drug Content (μg/mL)	
	Real-Time Stability Condition	Accelerated Stability Condition
0	99.99 ± 2.55	
1	96.45 ± 2.98	96.08 ± 6.75
4	94.90 ± 1.75	95.75 ± 3.54
8	95.61 ± 3.06	-
12	96.38 ± 1.04	-

Each value represents the mean of three experiments (*n* = 3) ± standard deviation.

**Table 4:** Pharmacokinetic parameters of pure drug (CC) powder, and CCSD_pM_ formulation (F1).

Formulations	CC	F1
*AUC* (h·ng/mL)	173.29 ± 30.27	771.87 ± 227.63 *
*C_max_* (ng/mL)	25.44 ± 6.28	188.75 ± 41.06 *
T_max_ (h)	0.83 ± 0.60	0.50 ± 0.27
t_1/2_ (h)	7.47 ± 2.81	4.93 ± 1.42
K_el_ (h^−1^)	0.10 ± 0.03	0.15 ± 0.05

Each value represents the mean of six experiments (*n* = 6), and the error bar represents the standard deviation. * *p* < 0.05 compared to free CC.

## Data Availability

The data presented in this study are available in the paper here.
